# Emerging roles of non-coding RNAs in fibroblast to myofibroblast transition and fibrotic diseases

**DOI:** 10.3389/fphar.2024.1423045

**Published:** 2024-07-24

**Authors:** Xuewu Xing, Scott A. Rodeo

**Affiliations:** ^1^ Department of Orthopaedics, Tianjin First Central Hospital, Tianjin, China; ^2^ Orthopedic Soft Tissue Research Program, Hospital for Special Surgery, New York, NY, United States

**Keywords:** non-coding RNAs, fibroblast, myofibroblast, fibrosis, therapies

## Abstract

The transition of fibroblasts to myofibroblasts (FMT) represents a pivotal process in wound healing, tissue repair, and fibrotic diseases. This intricate transformation involves dynamic changes in cellular morphology, gene expression, and extracellular matrix remodeling. While extensively studied at the molecular level, recent research has illuminated the regulatory roles of non-coding RNAs (ncRNAs) in orchestrating FMT. This review explores the emerging roles of ncRNAs, including microRNAs (miRNAs), long non-coding RNAs (lncRNAs), and circular RNAs (circRNAs), in regulating this intricate process. NcRNAs interface with key signaling pathways, transcription factors, and epigenetic mechanisms to fine-tune gene expression during FMT. Their functions are critical in maintaining tissue homeostasis, and disruptions in these regulatory networks have been linked to pathological fibrosis across various tissues. Understanding the dynamic roles of ncRNAs in FMT bears therapeutic promise. Targeting specific ncRNAs holds potential to mitigate exaggerated myofibroblast activation and tissue fibrosis. However, challenges in delivery and specificity of ncRNA-based therapies remain. In summary, ncRNAs emerge as integral regulators in the symphony of FMT, orchestrating the balance between quiescent fibroblasts and activated myofibroblasts. As research advances, these ncRNAs appear to be prospects for innovative therapeutic strategies, offering hope in taming the complexities of fibrosis and restoring tissue equilibrium.

## 1 Introduction

Fibroblast to myofibroblast transition (FMT) is a fundamental process that holds immense significance in various physiological contexts such as wound healing, tissue repair, and the pathogenesis of fibrotic diseases (Zhang et al., 2016; Usher et al., 2019; Blessing et al., 2021; Wang et al., 2023). This intricate transition is characterized by profound changes in cellular phenotype, encompassing alterations in cellular morphology, gene expression profiles, and the synthesis of extracellular matrix components (Michalik et al., 2018). These modifications collectively culminate in substantial tissue remodeling, which is essential for restoring tissue integrity and function following injury or damage (D'Urso and Kurniawan, 2020).

The process of FMT can be broadly divided into four stages (Wynn and Ramalingam, 2012). Initially, quiescent fibroblasts are activated in response to injury or stress signals, leading them to start proliferating. Following this, activated fibroblasts differentiate into myofibroblasts, characterized by the expression of alpha-smooth muscle actin (α-SMA) and increased production of extracellular matrix (ECM) components. Myofibroblasts then play a crucial role in extracellular matrix remodeling, depositing collagen and other ECM proteins to repair tissue. Normally, myofibroblasts undergo apoptosis once the tissue is repaired. However, in pathological conditions, myofibroblasts persist, leading to fibrosis. Several key signaling pathways regulate FMT (Zhang et al., 2023), including the Transforming Growth Factor-beta (TGF-β) pathway, which is a major driver of FMT, promoting myofibroblast differentiation and ECM production. The MAPK pathway is involved in fibroblast activation and differentiation, while the PI3K/Akt pathway plays a role in cell survival and proliferation during FMT.

The exploration of the molecular intricacies governing FMT has been a subject of extensive research, driven by the imperative to comprehend the underlying mechanisms that drive tissue repair and fibrosis (Li et al., 2015). In this context, recent scientific exploration has evaluated the pivotal role of non-coding RNAs (ncRNAs) as indispensable orchestrators of the FMT process (Zhang et al., 2023). Traditionally overlooked due to their lack of protein-coding capacity (Ilieva and Uchida, 2022), ncRNAs are now recognized as key players in shaping the delicate equilibrium between quiescent fibroblasts and their activated myofibroblast counterparts during FMT (Creemers and van Rooij, 2016).

The ensemble of ncRNAs, including microRNAs (miRNAs) (Lu and Rothenberg, 2018), long non-coding RNAs (lncRNAs) (Fernandes et al., 2019), and circular RNAs (circRNAs) (Li et al., 2018a), showcases a multifaceted array of regulatory molecules that converge to finely tune the transition from fibroblasts to myofibroblasts (Wang et al., 2014; Fan et al., 2021; Su et al., 2021). This cascade of molecular events encompasses miRNAs that function as fine-tuners (Miao et al., 2018), lncRNAs that orchestrate complex gene expression networks (Dong et al., 2022), and circRNAs that act as dynamic sponges and orchestrators of intricate interactions (Yang et al., 2022). These ncRNAs are far from being bystanders; rather, they intricately interweave with signaling pathways, transcription factors, and epigenetic modulators to steer the gene expression programs that govern FMT (Zhou et al., 2018; Niu et al., 2022; Hertig et al., 2023).

**FIGURE 1:**
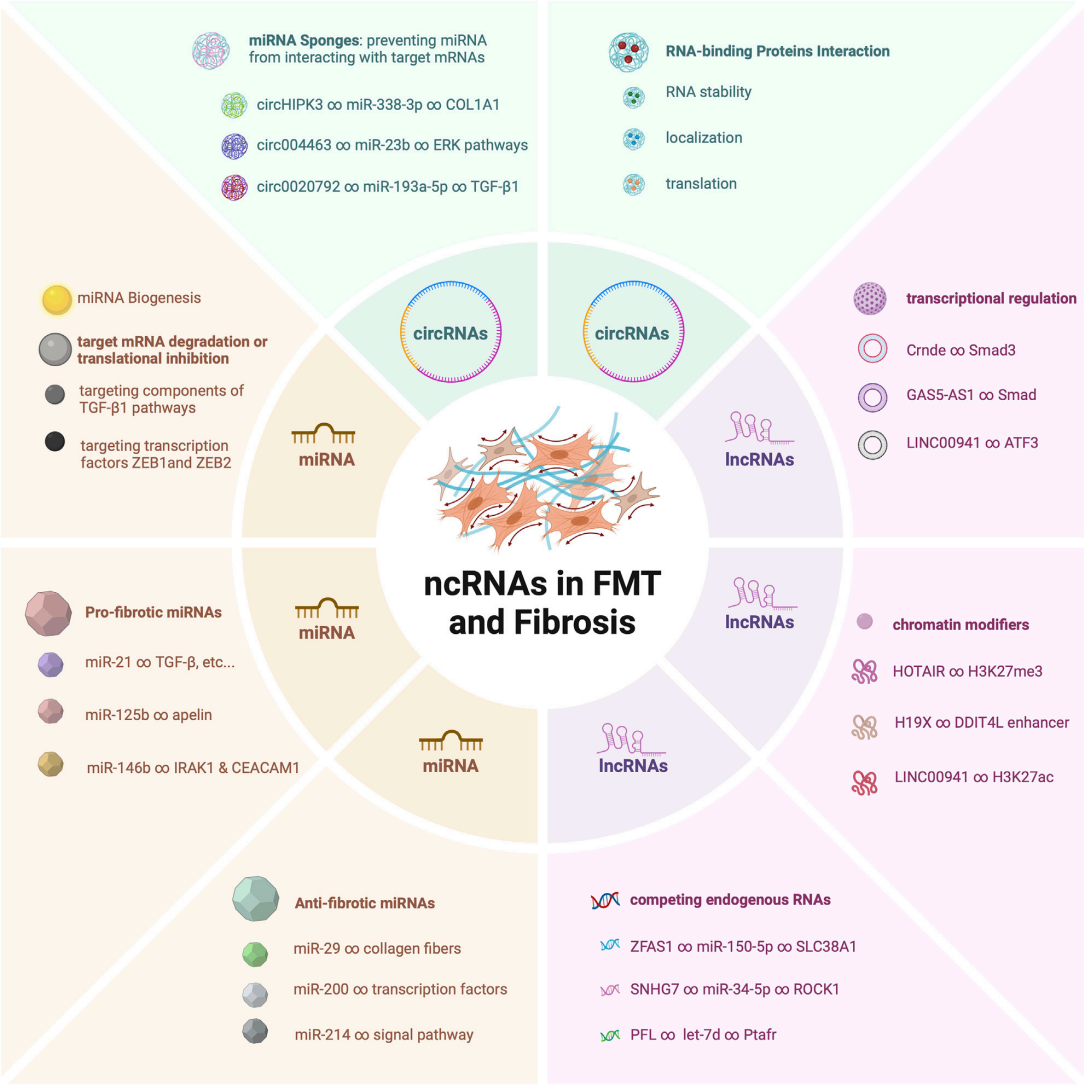
Mechanistic Roles of ncRNAs in Fibroblast-Myofibroblast Transition and Fibrosis.

The pivotal roles of these ncRNAs do not exist in isolation (Figure 1). Rather, they synergistically contribute to a complex regulatory network that dictates the fine balance between fibroblast quiescence and myofibroblast activation (Wang et al., 2020). Dysregulation of these ncRNAs has been found to be a common thread linking to the development of pathological fibrosis across diverse tissues (Tao et al., 2016; Tarbit et al., 2019; Senavirathna et al., 2020). Their dysregulated expression levels or altered interactions can have profound implications, leading to exaggerated myofibroblast activation (Wasson et al., 2020a), excessive extracellular matrix deposition (Zhang et al., 2018), and ultimately tissue fibrosis (Lino Cardenas et al., 2013).

In this review, our primary emphasis will be on elucidating the involvement of ncRNAs in both FMT process and fibrotic diseases, highlighting their significant therapeutic promise. Insights into their roles not only deepen our comprehension of fibrotic processes but also offer potential avenues for therapeutic interventions aimed at mitigating the excessive activation of myofibroblasts and inhibiting the progression of fibrosis.

## 2 MicroRNAs (miRNAs) in FMT

MicroRNAs (miRNAs) are a class of small non-coding RNA molecules, typically about 22 nucleotides in length, that play crucial roles in post-transcriptional gene regulation. MiRNAs exert their regulatory effects by binding to the 3’untranslated region (UTR) of target messenger RNAs (mRNAs), leading to mRNA degradation or translational repression. In the context of fibrotic diseases, miRNAs are significantly altered (Selman et al., 2016). Emerging evidence highlights the substantial impact of miRNAs in modulating FMT dynamics. MiRNAs play intricate roles in both promoting and inhibiting FMT, making them key regulators of this transition.

**FIGURE 2:**
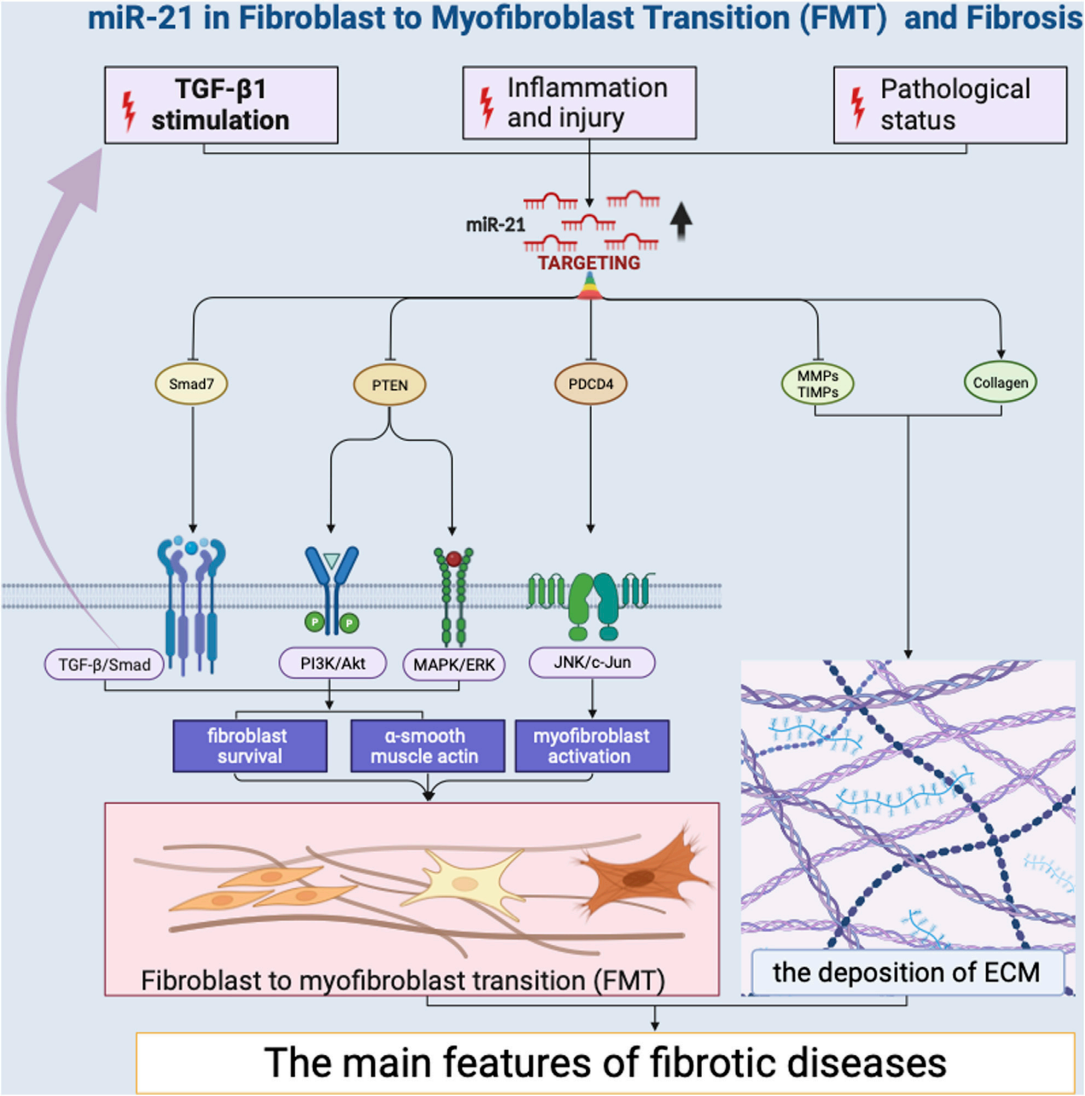
miR-21 in Fibroblast to Myofibroblast Transition (FMT) and Fibrosis.

Several miRNAs have been identified as promoters of FMT by targeting key regulators of the transition. Notably, miR-21 has emerged as a potent inducer of FMT (Liu et al., 2010; Yao et al., 2011; Liang et al., 2012; Wang et al., 2012; Bullock et al., 2013; Glowacki et al., 2013; Gong et al., 2014; Hedbäck et al., 2014; Lorenzen et al., 2015; Cui et al., 2018; Xu et al., 2018; Li et al., 2019; Kilari et al., 2019; Schipper et al., 2020; Wang et al., 2021; Nonaka et al., 2021; Ramanujam et al., 2021; Yang et al., 2021; Liao et al., 2022) (Figure 2). Its impact on FMT is primarily mediated through its ability to regulate the transforming growth factor-beta (TGF-β) signaling pathway (Liu et al., 2010; Yao et al., 2011; Liang et al., 2012; Cui et al., 2018; Nonaka et al., 2021; Yang et al., 2021). TGF-β is a pivotal cytokine that plays a central role in fibrotic processes (Fernandez and Eickelberg, 2012; Yousefi et al., 2020). MiR-21 achieves this regulatory effect by targeting TGF-β receptor inhibitors, leading to their downregulation. This downregulation results in an increased responsiveness of fibroblasts to TGF-β signaling, effectively priming them for myofibroblast differentiation. MiR-21 also promotes the expression of various extracellular matrix (ECM) components, such as collagens (Liang et al., 2012; Cui et al., 2018; Nonaka et al., 2021) and fibronectin (Cui et al., 2018), thereby contributing to the phenotypic shift of fibroblasts into myofibroblasts. This induction of ECM components strengthens the fibrotic matrix, leading to tissue remodeling and fibrosis development. Additionally, miR-146b have been shown to facilitate FMT by targeting interleukin 1 receptor-associated kinase 1 (IRAK1) and carcinoembryonic antigen-related cell adhesion molecule 1 (CEACAM1) that inhibit myofibroblast activation (Liao et al., 2021). The research revealed that miR-146b led to increased proliferation and migration of fibroblasts, the conversion of fibroblasts into myofibroblasts, and disrupted signaling among macrophages. Likewise, miR-125b contributes to FMT by downregulating apelin that would otherwise repress the activation of fibroblasts into myofibroblasts (Nagpal et al., 2016). This downregulation effectively removes barriers that restrain the transition process, resulting in enhanced myofibroblast formation. Collectively, these miRNAs exemplify the intricate regulatory landscape of FMT. Their effects extend beyond singular pathways, intertwining with the TGF-β, WNT, and PI3K/AKT signaling pathways (Zhang et al., 2023), ultimately driving the progression of fibrosis.

Conversely, certain miRNAs act as suppressors of FMT. These miRNAs play a crucial role in counteracting the signals and factors that drive fibroblasts towards myofibroblast differentiation, ultimately contributing to the maintenance of tissue homeostasis and preventing excessive fibrosis. The miR-29 family stands out as a group of miRNAs that counteract FMT by targeting collagen synthesis and deposition, essential processes in fibrosis (Kwiecinski et al., 2011; Wang et al., 2021; Yu et al., 2021; Yang et al., 2022; Xi et al., 2023). miR-29 directly targets and downregulates the expression of various collagens, including collagen type I (Yu et al., 2021), III (Wang et al., 2019), and IV (Kwiecinski et al., 2011), as well as other extracellular matrix components (Zhang et al., 2023). This regulatory mechanism orchestrated by miR-29 efficiently dampens the excessive accumulation of collagen fibers, which is a hallmark of fibrotic tissue remodeling. By inhibiting collagen production, miR-29 acts as a protection against the pathological transformation of fibroblasts into myofibroblasts, thus preventing the progression of fibrosis. Moreover, the miR-200 family members counteract FMT by targeting transcription factors ZEB1 (Bhome et al., 2022) and ZEB2 (Liao et al., 2018), which are integral to the epithelial-mesenchymal transition. By inhibiting ZEB1 and ZEB2 expression, miR-200 miRNAs effectively impede the transition of fibroblasts into myofibroblasts, contributing to the maintenance of the fibroblast phenotype and preventing fibrotic tissue remodeling. Similarly, miR-214 plays a role in inhibiting FMT by targeting factors that repress the activation of myofibroblasts (Izawa et al., 2015; Zhu et al., 2016; Yang et al., 2019). By suppressing these inhibitory elements, miR-214 helps tilt the balance in favor of myofibroblast differentiation. In brief, the balanced interplay between miRNAs that promote and those that suppress fibroblast to myofibroblast transition is crucial for maintaining tissue integrity and preventing pathological fibrosis. The opposing actions of these miRNAs create a finely tuned regulatory network that governs the dynamic equilibrium between fibroblasts and myofibroblasts.

In fibrotic conditions, miRNAs undergo various modifications that affect their expression and function. These modifications include changes in miRNA transcription, processing, and stability. Fibrotic signals such as TGF-β can induce or repress the transcription of specific miRNAs (Selman et al., 2016). Additionally, alterations in miRNA processing enzymes, such as Drosha and Dicer, can impact miRNA maturation and stability (Mishra et al., 2009; Cho et al., 2020). Epigenetic modifications, including DNA methylation and histone modifications, also play a role in regulating miRNA expression in fibrotic tissues (Yang et al., 2015). These mechanisms collectively contribute to the dysregulation of miRNAs in fibrosis, influencing their ability to modulate gene expression during FMT.

The regulatory roles of miRNAs in FMT are far from linear, as many miRNAs participate in intricate regulatory networks. MiRNAs often target multiple genes and pathways simultaneously, influencing the balance between pro-fibrotic and anti-fibrotic processes. This phenomenon allows miRNAs to fine-tune the overall outcome of FMT by modulating the expression of various genes that are involved in different stages of the transition. One key feature of miRNA-mediated regulation in FMT is the concurrent targeting of multiple genes within the same or related signaling pathways (Kwiecinski et al., 2011; Gong et al., 2014; Lorenzen et al., 2015; Nagpal et al., 2016; Wang et al., 2021; Medzikovic et al., 2023). This results in a synergistic impact on the cellular processes associated with FMT. This multi-targeting capacity enables miRNAs to exert a more potent and coordinated influence on FMT compared to a linear one-to-one relationship between miRNA and target gene. Cross-talk between miRNAs and other non-coding RNAs, such as lncRNAs(Li et al., 2018b; Wang et al., 2019) and circRNAs(Zhang et al., 2020; Ma et al., 2023), further complicates the regulatory landscape. The interplay between miRNAs, target genes, and other ncRNAs collectively constitutes a systems-level regulatory network that governs FMT. This network-based perspective highlights the interconnectedness and interdependence of various components in shaping the outcome of FMT.

MiRNAs play pivotal roles in orchestrating fibroblast to myofibroblast transition. Their dual nature as promoters and inhibitors of FMT underscores their complex regulatory functions in fibrosis. As our understanding of the roles of miRNAs in FMT continues to evolve, the prospects for innovative therapeutic strategies in fibrotic diseases become increasingly promising. The ability to manipulate miRNAs to finely tune the fibrotic response offers a level of precision that was previously unimaginable.

## 3 Long non-coding RNAs (lncRNAs) in FMT

Long Non-Coding RNAs (lncRNAs) constitute a diverse group of RNA molecules exceeding 200 nucleotides in length that lack protein-coding capacity but exert critical regulatory roles across various cellular processes. Within the intricate processes of FMT, a recent focus has emerged on lncRNAs as key regulatory elements. These lncRNAs establish their presence within the framework of FMT by orchestrating complex molecular interactions. They serve as regulators, directing the delicate interplay among chromatin modifiers, transcription factors, and a competing endogenous RNA (ceRNA) that govern gene expression patterns critical to FMT. These orchestrated activities assume a crucial role in the transformation of fibroblasts into myofibroblasts, a pivotal event in the development of tissue fibrosis. A diverse group of lncRNAs, including notable examples such as MALAT1 (Wu et al., 2015), H19X (Pachera et al., 2020), ZFAS1 (Yang et al., 2020), and SAFE (Hao et al., 2019), have garnered attention for their role as promoters of myofibroblast differentiation. Their contributions add a novel layer of regulatory intricacy to the evolving narrative surrounding FMT.

lncRNAs have emerged as key regulators of chromatin remodeling in the process of myofibroblast differentiation. These lncRNAs act as guides, directing chromatin modifiers to specific genomic loci that are strategically poised to undergo transformation. Through their interaction with chromatin-modifying complexes, these lncRNAs initiate a cascade of epigenetic changes that play a central role in the activation of genes critical for FMT. For example, HOTAIR and H19X have important effects on chromatin. Their strategic interaction with chromatin modifiers, including histone methyltransferases (Wasson et al., 2020b; Wang et al., 2023) and chromatin accessibility (Pachera et al., 2020), initiates the unwinding of the tightly packed chromatin structure. This allows for increased accessibility of transcription factors, such as GLI2, and other regulatory molecules to the gene promoters that drive myofibroblast differentiation. As chromatin remodeling takes place under the guidance of these lncRNAs, a series of events unfold that culminate in the activation of genes pivotal to FMT. These activated genes include those encoding extracellular matrix components, cytoskeletal proteins, and signaling molecules that are characteristic of the myofibroblast phenotype. The orchestrated chromatin changes initiated by lncRNAs lead to the establishment of a permissive transcriptional environment that favors the expression of genes essential for myofibroblast differentiation.

Through their intricate interplay with transcription factors smad, lncRNAs wield significant influence over the gene expression landscape that guides fibroblasts through the intricate process of myofibroblast differentiation. In zheng’s study (Zheng et al., 2019), Smad3 activated the expression of Crnde, revealing insights into the molecular process. Intriguingly, Crnde also suppressed Smad3’s transcriptional activation of target genes, thus blocking the expression of myofibroblast-specific marker genes in cardiac fibroblasts. Lin’s research demonstrated that GAS5-AS1 levels were significantly reduced in oral submucous fibrosis tissues and fibrotic buccal mucosal fibroblasts (Lin et al., 2018). Furthermore, increasing GAS5-AS1 expression led to inhibition of both p-Smad expression and myofibroblast markers. Their presence ensures the coordination, precision, and fidelity of gene expression programs essential for driving FMT. By functioning as transcriptional regulators, lncRNAs contribute to orchestrating a complex series of molecular events culminating in the acquisition of the myofibroblast phenotype.

In the realm of gene expression regulation, transcription factors assume the role of master regulators, directing the intricate sequence of molecular events that govern cellular differentiation. However, this role is not undertaken in isolation. lncRNAs serve as adept collaborators, guiding the transformative process of FMT. LncRNAs emerge as crucial co-regulators in this complex transcriptional symphony, intricately woven into the regulatory landscape to ensure the precise execution of gene expression programs that steer fibroblasts along the path of myofibroblast differentiation. Through specific interactions with transcription factors, they play a role beyond conventional transcriptional regulation. LINC00941 act as co-regulators, interacting transcription factors ATF3 and histone 3 lysine 27 acetylation to play its pro-fibrotic role (Zhang et al., 2022). This coordinated collaboration guarantees the timely and accurate activation of the genes necessary for driving the transformation of fibroblasts into myofibroblasts.

Furthermore, the co-regulator role of lncRNAs extends beyond mere guidance; LncRNA Airn actively participate in modulating the development of cardiac fibrosis via IMP2-p53 axis in an m6A dependent manner (Peng et al., 2022). Serving as molecular scaffolds, lncRNA H19X create a conducive environment for the assembly of complexes, thereby influencing the accessibility of target gene enhancer (Pachera et al., 2020). This interaction fine-tunes transcriptional activity, either amplifying or attenuating the expression of genes involved in FMT.

The incorporation of lncRNAs into the narrative of FMT introduces a fresh and intricate layer to the multifaceted story of fibrosis. These elusive molecules, previously overshadowed by protein-coding genes, now emerge as critical protagonists, orchestrating the delicate balance between fibroblast quiescence and the transformative process into myofibroblasts. By seamlessly integrating themselves into the complex molecular choreography of FMT, lncRNAs exert their influence in previously unforeseen ways. Their roles as guides, regulators, and network architects has provided new insights into our understanding of fibrosis, suggesting their potential as therapeutic targets for the benefit of patients afflicted with fibrotic conditions.

## 4 Circular RNAs (circRNAs) in FMT

Circular RNAs (circRNAs) are a class of non-coding RNAs (ncRNAs) characterized by their covalently closed loop structure. Unlike linear RNAs, circRNAs lack 5′caps and 3′polyadenylated tails, making them resistant to exonucleases. This unique structure imparts remarkable stability, allowing circRNAs to persist longer in cells compared to their linear counterparts (Kristensen et al., 2019). These stable molecules are involved in various cellular processes by acting as miRNA sponges, interacting with RNA-binding proteins, and influencing gene expression. In the intricate landscape of FMT, circRNAs have emerged as pivotal players, wielding their regulatory influence through multifaceted mechanisms that are now elucidated by recent studies.

One of the prominent roles that circRNAs play in FMT is that of miRNA sponges, implying that circRNAs have sequences that can bind to and interact with miRNAs, preventing them from carrying out their usual regulatory functions on other messenger RNAs. CircRNAs possess a remarkable ability to sequester miRNAs, small regulatory RNAs that modulate gene expression by binding to mRNA targets and suppressing their translation or promoting their degradation (Patop et al., 2019). By acting as miRNA sponges, circRNAs effectively titrate miRNAs away from their mRNA targets, thus preventing their inhibitory effects. This intricate regulation allows circRNAs to regulate gene expression programs that are crucial for FMT (Zhu et al., 2019; Hu et al., 2022; Zou et al., 2023). Notably, circRNAs like circHIPK3 have been identified as potent regulators of FMT-associated genes (Zhang et al., 2019). By binding to miR-338-3p, circHIPK3 prevents the miR-338-3p from interacting with their intended mRNA targets. As a result, the expression of target gene SOX4 and COL1A1, is spared from miRNA-mediated suppression, leading to the enhancement of fibroblast activation. This mechanism underscores the pivotal role circRNAs play in modulating gene expression patterns that drive the transition of fibroblasts into myofibroblasts.

Beyond their role as miRNA sponges, circRNAs also interact with RNA-binding proteins, adding another layer of complexity to their regulatory functions. For example, Circ-sh3rf3 (circular RNA SH3 domain containing Ring Finger 3) interacts with RNA-binding protein GATA-4 to promote the expression of miR-29a, thereby inhibiting FMT and myocardial fibrosis (Ma et al., 2023). These interactions can impact RNA stability, localization, and translation, further expanding the repertoire of mechanisms through which circRNAs influence FMT. Through their interactions with both miRNAs and RNA-binding proteins, circRNAs wield a dynamic and multifaceted influence on the regulatory networks that govern FMT.

Recent studies have also shed light on circRNAs’ role in modulating signaling pathways critical for FMT. CircTTN, for instance, has been implicated in the PI3K/AKT pathway, a key signaling cascade in myofibroblast differentiation. By sponging miR-432, circTTN regulates the expression of genes like IGF2, thereby influencing the activation of the PI3K/AKT signaling pathway (Wang et al., 2019). This regulation demonstrates how circRNAs can modulate specific signaling pathways, affecting the cellular transitions in fibrosis.

Furthermore, circRNAs like circ004463 have been found to interact with AKT/ERK pathways. Circ004463 sponges miR-23b, which targets the mRNA of AKT and ERK. By regulating CADM3 and MAP4K4 expression, circ004463 plays a significant role in promoting fibroblast proliferation and collagen type I synthesis (Zou et al., 2023). Another notable circRNA is hsa_circ_0020792, which acts as a sponge for miR-193a-5p, thereby regulating the expression of pro-fibrotic genes such as TGF-β1 (Hu et al., 2022). This interaction is crucial in the context of fibrosis, as TGF-β1 is a key cytokine driving fibrogenesis, and collagen type I is a major component of the extracellular matrix.

The intricate regulatory function of circRNAs within the context of FMT suggests their significance in shaping cell fate. Their capacity to sponge miRNAs and interact with RNA-binding proteins underscores their ability to modulate gene expression programs, thus determining whether fibroblasts remain in their quiescent state or transition into myofibroblasts. As ongoing research unravels the intricacies of these regulatory mechanisms, circRNAs hold the promise of becoming not only diagnostic markers but also potential therapeutic targets for mitigating the progression of fibrotic diseases.

## 5 Role of non-coding RNAs (ncRNAs) in fibrotic diseases

Recent research reveals a substantial exploration into the contribution of dysregulated ncRNAs to the intricate landscape of pathological fibrosis. These ncRNAs have emerged as critical players in driving the development and progression of fibrotic diseases across diverse tissues. MiRNAs exhibit a multifaceted role in pathological fibrosis. Pro-fibrotic miRNAs, exemplified by miR-21, facilitate fibrosis by augmenting fibroblast responsiveness to profibrotic stimuli and promoting extracellular matrix deposition. Conversely, anti-fibrotic miRNAs like miR-133a (Wei et al., 2019), counteract fibrosis by targeting multiple components of TGF-β1 profibrogenic pathways. LncRNAs exert significant influence on pathological fibrosis. Pro-fibrotic lncRNAs such as HOTAIR and H19X contribute to myofibroblast differentiation by engaging with chromatin modifiers, transcription factors, and regulatory molecules. This interaction modulates gene expression profiles and drives fibroblasts towards the myofibroblast phenotype. In contrast, certain lncRNAs such as PFI (Sun et al., 2021) and LOC344887 (Liu et al., 2021) act as suppressors of fibrosis, impeding myofibroblast activation and promoting tissue equilibrium. CircRNAs, with their circular structure, introduce an additional layer of complexity to the fibrotic scenario. Operating as miRNA sponges and interacting with RNA-binding proteins, circRNAs regulate gene expression patterns with precision. CircRNAs like circHIPK3 exemplify this role by sequestering miRNAs targeting key genes involved in fibrotic processes, thereby modulating gene expression profiles that underpin fibrosis. In summary, prior studies underscore the integral roles of dysregulated ncRNAs in driving pathological fibrosis. These ncRNAs impact the equilibrium between fibroblast activation and tissue health.

The formation and expression of ncRNAs are tightly regulated processes that are often altered during disease conditions. ncRNAs are transcribed by RNA polymerase II and III, and their maturation involves complex processing steps, including splicing, editing, and modifications. For example, primary miRNAs (pri-miRNAs) are processed by Drosha and Dicer enzymes to generate mature miRNAs that can bind to target mRNAs (Herrera et al., 2018; Cho et al., 2020). Similarly, lncRNAs undergo splicing and modifications that influence their stability and function (Hao et al., 2019). The expression of ncRNAs is tightly regulated under normal conditions but can become dysregulated during fibrosis. This dysregulation plays a crucial role in the pathological progression of fibrosis by affecting the balance between fibroblast quiescence and myofibroblast activation. In kidney fibrosis, the upregulation of miR-21 correlates with increased kidney stiffness and fibrosis severity, indicating its role in disease progression (Glowacki et al., 2013). Similarly, reduced levels of miR-449a are observed in fibrotic lung tissues and correlate with the severity of lung lesions induced by silica, suggesting its involvement in the Silicosis (Han et al., 2016). Understanding the correlation between ncRNA expression and fibrosis progression provides valuable insights into the molecular mechanisms underlying fibrotic diseases. These insights highlight the potential of ncRNAs as biomarkers for disease diagnosis and prognosis and as therapeutic targets for modulating fibrotic processes and restoring tissue homeostasis.

Notably, ncRNAs exhibit their multifaceted roles across a diverse spectrum of fibrotic conditions, ranging from cardiac fibrosis, hepatic fibrosis, pulmonary fibrosis, renal fibrosis, dermal fibrosis, and musculoskeletal tissues (Table 1). This broad influence underscores the significance of ncRNAs as central regulators of fibrotic processes across diverse tissues and organs. In particular, arthrofibrosis is a common and debilitating complication that can occur following knee surgery (Lee et al., 2022). Abdel et al. have identified differentially expressed genes associated with arthrofibrosis by comparing tissue samples from fibrotic and non-fibrotic human knee joints using RNA sequencing (Bayram et al., 2020). Further, Chen et al. carried out further bioinformatics analysis and reported new biomarkers for diagnosing arthrofibrosis, shedding light on the role of transforming growth factor-beta receptor 1 (TGFBR1) (Chen et al., 2021). These data provide further insight into the role of ncRNAs in the regulation of joint fibrosis.

**TABLE 1:** ncRNAs spectrum of diverse fibrotic diseases.

	miRNAs	lncRNAs	circRNAs
cardiac fibrosis	miR-9 (Wang et al., 2016c), miR-21 (Liang et al., 2012; Lorenzen et al., 2015; Nonaka et al., 2021; Ramanujam et al., 2021), miR-22 (Zhang et al., 2018b), miR-23a-3p (Su et al., 2022), miR-29 b (Horii et al., 2023), MiR-32–5p (Shen et al., 2019), miR-34a/miR-93 (Zhang et al., 2018a), miR-101a (Zhou et al., 2018), miR-125 b (Nagpal et al., 2016; Dufeys et al., 2021), miR-130a (Li et al., 2017a; Feng et al., 2022), miR-133a (Matkovich et al., 2010), miR-135a (Wei et al., 2020), miR-142–3p (Wang et al., 2016d; Cai et al., 2020a), miR-150 (Deng et al., 2016), miR-152–3p (Xu et al., 2021b), miR-155 (Zhang et al., 2016b; Wei et al., 2017), miR-195–3p (Carvalho et al., 2023), miR-214–3p (Zhu et al., 2016; Yang et al., 2019), miR-216a (Qu et al., 2019), miR-327 (Ji et al., 2018), miR-331 (Yousefi et al., 2021), miR-338–3p (Huang et al., 2022), miR-369–5p (Tao et al., 2018), miR-409–3p (Wang et al., 2022), miR-433 (Tao et al., 2016b), miR-451a (Deng et al., 2022), miR-486 (Chen et al., 2022), miR-574–5p (Cui et al., 2020)	Airn (Peng et al., 2022) Crnde (Zheng et al., 2019) Gm41724 (Kong et al., 2023) PFL (Liang et al., 2018) RMST (Ma et al., 2023b) Safe (Hao et al., 2019) SRA1(Zhang et al., 2019c) SNHG7(Wang et al., 2020b) TUG1 (Zhu et al., 2018)	circNFIB(Zhu et al., 2019) circHRCR (Wang et al., 2016b) circ-sh3rf3 (Ma et al., 2023a) circSMAD4 (Jeong et al., 2023)
pulmonary fibrosis	let-7 (Elliot et al., 2019; Thakur et al., 2022; Xu et al., 2022), miR-7 (Zhang et al., 2020b), miR-9-5p (Fierro-Fernández et al., 2015), miR-19a (Fujita et al., 2023), miR-21 (Yamada et al., 2013; Cui et al., 2018; Wang et al., 2021a), miR-22 (Kuse et al., 2020), miR-24 (Ebrahimpour et al., 2019), miR-26a (Liang et al., 2014), miR-27a-3p (Cui et al., 2016), miR-29 (Herrera et al., 2018), miR-30c (Kanno et al., 2021), miR-30d (Zhao et al., 2018), miR-34a (Cui et al., 2017; Bulvik et al., 2020), miR-34b-5p (Hu et al., 2019), miR-96 (Nho et al., 2014), miR-124 (Lu et al., 2019), miR-133a (Wei et al., 2019), miR-144–3p (Bahudhanapati et al., 2019), miR-145 (Yang et al., 2013), miR-155 (Artlett et al., 2017), miR-199a-5p (Lino Cardenas et al., 2013; Yi et al., 2018), miR-200 (Chilosi et al., 2017), miR-338–3p (Rackow et al., 2022), miR-424 (Xiao et al., 2015; Huang et al., 2020), miR-375 (Zhang et al., 2020c), miR-449a (Han et al., 2016), miR-497–5p (Chen et al., 2017), miR-541–5p (Ren et al., 2017), miR-627 (Li et al., 2019a), miR-877–3p (Wang et al., 2016a), miR-7219–3p (Niu et al., 2022)	CTD-2528L19.6 (Chen et al., 2021a) DNM3OS(Savary et al., 2019) GAS5 (Wang et al., 2023b) H19 (Xiao et al., 2021) ITPF(Song et al., 2019) LINC00941(Zhang et al., 2022) LOC344887(Liu et al., 2021) LOC103691771(Cai et al., 2020b) PFI(Sun et al., 2021) PFAL(Li et al., 2018b) SNHG1(Wu et al., 2021) SNHG20(Cheng et al., 2021) ZFAS1(Yang et al., 2020)	circ0044226 (Zhang et al., 2020a) circHIPK3(Zhang et al., 2019b; Xu et al., 2021a)
renal fibrosis	miR-34a (Saito et al., 2023), miR-132 (Bijkerk et al., 2016), miR-335–5p (Qiu et al., 2022), miR-378a-5p (Zhang et al., 2023b)	Rian and Miat (Bijkerk et al., 2019)	—
hepatic fibrosis	miR-16 (Pan et al., 2020), miR-19 b (Brandon-Warner et al., 2018), miR-29 (Kwiecinski et al., 2011; Kwiecinski et al., 2012), miR-132 (Mann et al., 2010), miR-214 (Izawa et al., 2015)	MALAT1 (Wu et al., 2015)	—
dermal fibrosis	miR-130a (Zhang et al., 2019a), miR-192 (Li et al., 2017b; Li et al., 2021), miR-196b-5p (Baral et al., 2021)	HOTAIR (Wasson et al., 2020b)	circAMD1 (Su et al., 2021)
oral submucous fibrosis	miR-10 b (Fang et al., 2020), miR-21 (Yang et al., 2021; Liao et al., 2022), miR-29c (Yang et al., 2022a), miR-200 b (Liao et al., 2018)	GAS5-AS1 (Lin et al., 2018) HOTTIP(Lee et al., 2021) H19 (Yu et al., 2021)	—
musculoskeletal tissues	miR-29a (Millar et al., 2015),miR-214–3p (Arrighi et al., 2021)	—	circTTN (Wang et al., 2019b)

ncRNAs exhibit both ubiquitous and tissue-specific functions, which together shape the initiation and progression of fibrosis. Ubiquitous ncRNAs, such as miR-21, are widely expressed across different tissues and play a central role in fibrosis by modulating common fibrogenic pathways. miR-21 enhances fibroblast activation and extracellular matrix deposition by targeting multiple genes involved in the TGF-β signaling pathway, including SMAD7 and PTEN, thus promoting fibrosis in various organs (Glowacki et al., 2013; Li et al., 2019; Wang et al., 2021; Nonaka et al., 2021; Liao et al., 2022). In contrast, tissue-specific ncRNAs are expressed in particular organs and contribute to localized fibrotic processes. For instance, lncRNA MALAT1 is prominently expressed in the liver and contributes to hepatic fibrosis by interacting with the silent information regulator 1(SIRT1) and promoting the expression of pro-fibrotic genes such as COL1A1 and α-SMA (Wu et al., 2015). Similarly, circNFIB is predominantly expressed in the heart and, where it activates the TGF-β–Smad3 signaling pathway and is crucial in cardiac fibrosis (Zhu et al., 2019).

ncRNAs can exert paracrine effects, influencing cells beyond their origin and contributing to multi-organ fibrosis. These ncRNAs can be secreted into the extracellular environment and transported to distant cells and tissues through extracellular vesicles (EVs), such as exosomes and microvesicles. This capability allows ncRNAs to participate in intercellular communication and influence various physiological and pathological processes across different organs.

In the context of fibrosis, ncRNAs can be secreted by fibroblasts or other cell types and taken up by neighboring cells, thereby modulating their behavior. For example, miR-21, a well-known pro-fibrotic miRNA, can be packaged into EVs and transferred from myofibroblasts to adjacent endothelial cells. This transfer can induce a pro-angiogenic process of endothelial cells, a process contributing to the fibrotic response (Li et al., 2019). Similarly, miR-200, another miRNA implicated in fibrosis, can be secreted by endothelial cells and taken up by fibroblasts, influencing fibroblast heterogeneity in colorectal cancer (Bhome et al., 2022).

NcRNAs can enter the systemic circulation, allowing them to travel to distant organs and exert their effects. Circulating miRNAs, for instance, have been detected in blood, urine, and other body fluids, serving as biomarkers for various diseases (De Guire et al., 2013). These circulating ncRNAs extend their impact beyond the local tissue environment, affecting distant organs and contributing to the pathology of multi-organ diseases. For instance, miR-29, which regulates extracellular matrix production, is involved in cutaneous, prostate, cardiac and oral submucous fibrosis. Its dysregulation in one organ can have implications for fibrotic processes in others.

LncRNAs also exhibit multi-organ effects. LncRNA H19, known for its role in pulmonary fibrosis, can influence fibrotic buccal mucosal myofibroblast activities, such as collagen gel contractility and migration ability when dysregulated, highlighting its potential impact on both pulmonary and oral submucous tissues. Similarly, the lncRNA GAS5, which modulates fibrotic pathways in the skin, can have systemic effects, potentially affecting other fibrotic conditions in organs like the lung.

Understanding the paracrine and multi-organ effects of ncRNAs is crucial for developing therapeutic strategies targeting fibrotic diseases. Therapies designed to modulate ncRNA levels in one organ might have beneficial effects on fibrosis in other organs, offering a systemic approach to treating multi-organ fibrotic conditions. For example, therapeutic inhibition of miR-21 has shown promise in reducing fibrosis in both the heart and lung, demonstrating the potential of ncRNA-targeted therapies to address multi-organ fibrosis.

ncRNAs may have distinct impacts on acute versus chronic diseases, reflecting their roles in immediate injury responses versus long-term maladaptive processes. During acute injury, the rapid and transient changes in ncRNA expression are crucial for the immediate response to cellular damage and stress. For instance, miR-101a is rapidly upregulated following myocardial infarction (MI) and plays a critical role in promoting cardiac fibroblast activation and fibrosis to stabilize the injured tissue (Zhou et al., 2018). In contrast, chronic conditions and aging involve sustained ncRNA dysregulation, contributing to persistent fibrosis and organ dysfunction. For example, miR-34A is consistently dysregulated in chronic liver and renal fibrosis, leading to sustained extracellular matrix production and fibrogenesis (Cui et al., 2017; Saito et al., 2023). Understanding the distinct roles of ncRNAs in acute and chronic conditions can inform the development of targeted therapies. In acute injury, therapeutic strategies may aim to modulate ncRNAs to enhance tissue repair and limit damage. In chronic diseases and aging, ncRNA-based therapies could focus on reversing maladaptive gene expression patterns and reducing fibrosis and inflammation.

In short, the regulatory influence of various ncRNAs extends across diverse fibrotic diseases. The pervasive presence of these ncRNAs within the fibrotic milieu underscores the need for a comprehensive understanding of their intricate functions. Unraveling the precise molecular mechanisms through which ncRNAs exert their regulatory effects could pave the way for the development of targeted therapeutic strategies. By targeting these ncRNAs or modulating their interactions with key regulatory molecules, it might be possible to attenuate fibrosis progression and restore tissue homeostasis in a range of fibrotic diseases.

## 6 Therapeutic implications

The intricate involvement of ncRNAs in FMT has opened new avenues for therapeutic interventions in fibrotic diseases. These regulatory molecules have been identified as critical players in fine-tuning gene expression programs that govern the delicate balance between fibroblast quiescence and myofibroblast activation. By deciphering the precise roles of ncRNAs in regulating this transition, researchers have uncovered potential targets that could be manipulated to mitigate the excessive activation of myofibroblasts and slow the progression of fibrosis.

Therapeutic strategies involving miRNAs typically include miRNA mimics to restore the function of downregulated miRNAs or miRNA antagonists (antagomirs) to inhibit the function of upregulated miRNAs. For instance, MiR-29 family mimics exhibit antifibrotic effects across various tissues by targeting collagen synthesis and extracellular matrix remodeling. A completed open-label phase 2 RCT clinical trial (Clinical Trial Number: NCT03601052) has defined the efficacy, safety, and tolerability of Remlarsen (MRG-201), which is designed to mimic the activity of miR-29 that may be an effective therapeutic to prevent cutaneous fibrosis. This study demonstrated that administering high doses of this miR-29 mimic could effectively decrease fibrosis (Gallant-Behm et al., 2019). It is worth noting that the dosage utilized in this research was excessively high for practical use in human patients. Nevertheless, these findings provided encouraging evidence for investigators working towards the development of microRNA mimics as potential therapeutics for fibrosis. Anti-miR oligonucleotides, designed to inhibit the function of pro-fibrotic miRNAs, also show potential; for example, targeting miR-21, a pro-fibrotic miRNA, has shown promise in reducing fibrosis in preclinical models. Anti-miR-21 therapies aim to decrease fibroblast responsiveness to pro-fibrotic stimuli and reduce extracellular matrix deposition.

LncRNA-based therapeutics involve targeting pro-fibrotic lncRNAs, such as ASLNCS5088 (Chen et al., 2019) and Gm41724 (Kong et al., 2023), to mitigate fibrosis by disrupting their interactions with RNA-binding proteins, and M2 macrophage modulation. By preventing these interactions, it is possible to modulate gene expression profiles that drive fibroblast activation and myofibroblast differentiation. Additionally, boosting the expression of anti-fibrotic lncRNAs like GAS5 can help inhibit myofibroblast activation and fibrogenesis through suppressing TGF-β/Smad3 signaling (Tang et al., 2020). Therapeutic strategies may involve gene therapy approaches to deliver these lncRNAs or small molecules that enhance their endogenous expression.

CircRNA-based therapeutics focus on the unique abilities of circRNAs to act as miRNA sponges or interact with RNA-binding proteins. CircHIPK3 serves as a prime example, as it influences myofibroblast differentiation by sponging miR-338-3p that target SOX4 and COL1A1 (Zhang et al., 2019). Designing synthetic circRNA sponges can regulate miRNA activity and modulate gene expression patterns involved in fibrosis. Additionally, modulating circRNA-protein interactions can impact the regulatory networks driving fibrosis. For example, Circ-sh3rf3 can bind to GATA-4 proteins and decrease their expression, which prevents GATA-4 from suppressing miR-29a expression. As a result, miR-29a expression is increased, leading to the inhibition of fibroblast-to-myofibroblast differentiation and myocardial fibrosis. Targeting these ncRNAs might offer a means to disrupt the regulatory networks that drive fibroblast activation. Such precision-based approaches could revolutionize the treatment landscape for fibrotic diseases, allowing for tailored interventions that target the underlying molecular mechanisms.

While the potential of ncRNA-based therapies for fibrosis is exciting, several challenges must be navigated for successful translation into clinical applications. One significant hurdle is the delivery of ncRNA-based therapeutics to target tissues. Ensuring efficient and specific delivery remains a key obstacle. Strategies such as viral vectors (Tang et al., 2020), nanoparticle-mediated delivery (Zahir-Jouzdani et al., 2018), or organ-targeted liposomes (Yan et al., 2023) are being explored to address this challenge. Additionally, the specificity of ncRNA-targeting therapies is crucial to avoid off-target effects and unintended consequences (Yan et al., 2023). Ensuring that therapies selectively target the dysregulated ncRNAs while preserving the physiological functions of others is essential for clinical success. The stability and bioavailability of ncRNA-based therapeutics are critical factors for their effectiveness. Chemical modifications, such as locked nucleic acids (LNAs) and phosphorothioate backbones, can enhance the stability and resistance of ncRNA-based therapeutics to degradation. These modifications improve the pharmacokinetic properties and therapeutic efficacy of ncRNA-based treatments (Ali Zaidi et al., 2023). ncRNA-based therapeutics, particularly those involving viral vectors, may elicit immune responses. Strategies to minimize immunogenicity include optimizing vector design, using tissue-specific promoters, and developing non-viral delivery systems (Awan et al., 2017). Furthermore, the complex regulatory networks involving ncRNAs add another layer of complexity. Many ncRNAs participate in intricate crosstalk with other regulatory molecules, such as transcription factors and signaling pathways, leading to a network of interdependencies. Designing therapies that effectively modulate these networks requires a deep understanding of the molecular interactions and their consequences.

Future directions and prospects in ncRNA-based therapies for fibrotic diseases include combination therapies, personalized medicine, advancements in delivery systems, and robust translational research efforts. Combining ncRNA-based therapies with existing antifibrotic drugs or other therapeutic modalities may enhance efficacy and overcome resistance mechanisms. Personalized approaches tailored to individual patients’ specific ncRNA expression profiles can improve treatment outcomes and minimize adverse effects. Ongoing advancements in delivery systems, such as exosome-based delivery and tissue-specific nanoparticles, hold promise for improving the targeted delivery of ncRNA-based therapeutics. Collaborative efforts between academia, industry, and regulatory agencies can accelerate the development and approval of ncRNA-based therapies.

The emerging roles of ncRNAs in FMT offer novel avenues for therapeutic intervention in fibrotic diseases. By targeting specific ncRNAs, it is possible to intervene in the processes that drive myofibroblast activation and tissue fibrosis. However, the journey from bench to bedside requires the successful resolution of delivery challenges, the mitigation of off-target effects, and in depth understanding of the complex regulatory networks involved. As research in this field advances, the development of effective and precise therapies holds the promise of transforming the landscape of fibrotic disease treatment.

## 7 Conclusion

In summary, recent research highlights the crucial involvement of ncRNAs in the complex process of FMT. These ncRNAs, including miRNAs, lncRNAs, and circRNAs, collectively constitute a regulatory ensemble that finely modulates the equilibrium between quiescent fibroblasts and their activated myofibroblast counterparts. Recent studies have meticulously unraveled the multifaceted mechanisms by which these ncRNAs exert their influence.

The narrative begins with miRNAs, which play a central role by targeting key regulators of FMT. MiR-21 assumes a prominent position as a potent inducer of FMT, primarily by inhibiting TGF-β receptor inhibitors. This action sensitizes fibroblasts to TGF-β signaling, thereby promoting myofibroblast differentiation and subsequent fibrosis. MiR-146b and miR-125b also contribute to FMT by targeting factors that otherwise restrain myofibroblast activation. Conversely, miRNAs such as miR-29 and the miR-200 family act as suppressors of FMT, counteracting excessive collagen synthesis and inhibiting myofibroblast differentiation through their targeting of related genes.

LncRNAs act as pivotal regulators of FMT. Notable lncRNAs like H19X and GAS5 emerge as regulators in the FMT process. They engage with chromatin modifiers, transcription factors, and regulatory molecules, facilitating chromatin remodeling, reprogramming of gene expression, and the orchestration of transcriptional forces that guide fibroblasts toward the myofibroblast lineage. LncRNAs further their influence by fostering crosstalk among regulatory molecules, perpetuating essential signaling cascades crucial for FMT progression.

Simultaneously, circRNAs embrace their role as miRNA sponges, intricately fine-tuning gene expression during FMT. Notable circRNAs like circHIPK3 demonstrate their ability to sequester miRNAs targeting genes associated with FMT. In doing so, these circRNAs release these genes from miRNA-mediated suppression, ultimately enhancing the differentiation of fibroblasts into myofibroblasts. Moreover, the intricate interactions of circRNAs with RNA-binding proteins add an additional layer of complexity to their regulatory repertoire.

In a broader context, these ncRNAs collaboratively interweave their actions, constructing a complex network of regulatory interactions that modulate the transformation of fibroblasts into myofibroblasts. Their contributions extend beyond individual roles, creating a dynamic interplay that profoundly influences the delicate equilibrium between fibroblast quiescence and myofibroblast activation. Dysregulation of these ncRNAs has been closely linked to the development of pathological fibrosis in various tissues, underscoring their significance as potential therapeutic targets.

As we stand on the cusp of a new era in the treatment of fibrotic diseases, the emerging roles of ncRNAs in FMT offer substantial therapeutic promise. By deciphering the intricacies of ncRNA-mediated regulatory networks, researchers could uncover innovative therapeutic avenues that could effectively counteract the progression of fibrotic diseases. However, translating these insights into clinical applications presents challenges such as efficient delivery methods, specificity, and the potential for off-target effects. As the journey continues, the potential to harness the power of ncRNAs may illuminate a path toward restoring tissue health and function, offering renewed hope to those affected by these debilitating conditions.

In conclusion, the process of FMT occupies a central role in tissue repair and the pathogenesis of fibrotic diseases. The intricate interplay of cellular morphological changes, altered gene expression profiles, and extracellular matrix remodeling underscores its significance. With recent discoveries revealing the pivotal roles of ncRNAs, including miRNAs, lncRNAs, and circRNAs, in orchestrating FMT, a new chapter has opened in our understanding of tissue remodeling. These ncRNAs act as master regulators, shaping the symphony of FMT by influencing a diverse array of molecular players. Their regulatory capabilities extend across signaling cascades, transcriptional programs, and intricate interactions, and their dysregulation can lead to pathological fibrosis. As research continues to elucidate the precise mechanisms by which ncRNAs guide FMT, their therapeutic potential emerges as a promising frontier, offering novel strategies to combat fibrotic diseases and restore tissue health.
